# MiR‐372‐3p promotes cell growth and metastasis by targeting *FGF9* in lung squamous cell carcinoma

**DOI:** 10.1002/cam4.1026

**Published:** 2017-04-24

**Authors:** Qing Wang, Siyang Liu, Xitong Zhao, Yuan Wang, Dali Tian, Wenjun Jiang

**Affiliations:** ^1^ Department of Thoracic SurgeryThe Fourth Affiliated Hospital of China Medical UniversityShenyangLiaoning110032China

**Keywords:** FGF9, LSCC, MiR‐372‐3p

## Abstract

The aim of this study was to study the role of miR‐372‐3p in lung squamous cell carcinoma (LSCC) cell proliferation and invasion by suppressing *FGF9*. RT‐PCR was used to determine miR‐372‐3p and *FGF9* mRNA expression in tissues and cells. Western blot was used to determine FGF9 expression in tissues and NCI‐H520 cell line. Dual luciferase reporter gene assay was conducted to confirm that *FGF9* can be directly targeted by miR‐372‐3p. MTT, colony formation assays were conducted to investigate the effects of ectopic miR‐372‐3p and *FGF9* expression on NCI‐H520 cell growth. Flow cytometry was used to analyze the influence of miR‐372‐3p and FGF9 expression on cell cycle distribution and apoptosis. Transwell assay was also conducted to see the effects of miR‐372‐3p and FGF9 expression on NCI‐H520 cell invasiveness. MiR‐372‐3p was found significantly overexpressed in both LSCC tissues and cell lines, whereas *FGF9* mRNA was found underexpressed in LSCC tissues. MiR‐372‐3p directly bound to wild‐type *FGF9* mRNA 3′UTR, therefore led to the reduction in *FGF9* expression. The upregulation of FGF9 or the downregulation of miR‐372‐3p substantially retarded LSCC cell growth, mitosis, and invasion. MiR‐372‐3p enhanced LSCC cell proliferation and invasion through inhibiting *FGF9*.

## Introduction

Lung cancer is the principal cause of deaths related to cancer worldwide 1. Non‐small‐cell lung cancers (NSCLC) account for 80% of lung cancer, and are classified into four subtypes according to their histological and pathological features: lung adenocarcinoma, lung squamous cell carcinoma (LSCC), lung large cell carcinoma, and lung neuroendocrine cancer 2, 3. Approximately 40% of patients with NSCLC that are at advanced stage hardly survive more than 5 years despite of traditional chemotherapy, molecular‐targeted therapy or chemoradiotherapy 2, 3, 4. Therefore, novel effective treatments are demanded to tackle the short survival time of NSCLC.

The abnormal activation of fibroblast growth factor (FGF) signaling pathways is required for tissue homeostasis, repair, angiogenesis, organogenesis, and is involved in carcinogenesis 5, 6, 7, 8, 9, 10, indicating its potential role of a target for therapeutic intervention 5, 10, 11. By interacting with FGF receptors (FGFRs) and FGF ligands, FGFs modulate cell proliferation, differentiation and mobility 9. *FGF9*, belonging to the FGF family, its aberrant expression has been identified in diverse tumors, such as breast, prostate, endometrioid, and lung cancers, indicating its tumor biomarker role in various cancers 12, 13, 14, 15. Furthermore, Wang et al. indicated that *FGF9* was upregulated in patients with lung adenocarcinoma, and that aberrant *FGF9* expression might inhibit the progression of lung adenocarcinoma 16. However, little is known about the role of *FGF9* during LSCC.

MiRNAs, a class of small single strand RNA molecules, modulate the expression of approximately a third of human genes, and affect diverse cellular activities such as cell proliferation, cycle, apoptosis, and differentiation 17. Circulating miRNAs have great potential to serve as diagnostic/prognostic biomarkers for diverse cancers, such as gastric, bladder, breast, prostate, renal, and lung cancers 18, 19, 20, 21, 22, 23. MiRNA‐371‐373 (miR‐371‐373) cluster, a human homolog of the mouse miR‐290‐295 cluster is initially reported to be merely expressed in human embryonic stem cells, implicating its role in stem cell multipotency 24. Recent studies have also confirmed that the miR‐371‐373 cluster is usually deregulated in human tumors such as hepatoblastoma, colorectal cancer, and testicular germ cell tumors 25, 26, 27. MiRNAs function as tumor suppressors or tumor facilitators by directly binding to target 3′UTRs. MiR‐373, for instance, has been found to facilitate tumor invasion and metastasis by inhibiting CD44 28. We herein conducted this study to explore the role of miR‐372‐3p in LSCC.

In this study, we identified *FGF9* as a target gene of miR‐372‐3p. We also confirmed a converse correlation between miR‐372‐3p and *FGF9* in LSCC tissues and cells. The direct suppression of *FGF9* by miR‐372‐3p and the potential effects of miR‐372‐3p as a tumor facilitator in LSCC have been validated at both experimental and clinical levels.

## Materials and Methods

### Clinical samples

Twenty LSCC and corresponding adjacent normal tissues were obtained from those underwent pneumonectomy from April 2015 to December 2016 in The Fourth Affiliated Hospital of China Medical University. The pathological type of each tumor sample was confirmed by experienced pathologists. Fresh samples were frozen in the liquid nitrogen prior to RNA extraction. None of the patients had ever received any adjuvant chemotherapy or radiotherapy before surgery. Informed consent was signed by every patient. The study was approved by the Ethical Committee of The Fourth Affiliated Hospital of China Medical University.

### Cell culture

BEAS‐2B, a normal lung epithelial cell line, together with three human LSCC cell lines including NCI‐H520, SK‐MES‐1, and NCI‐H1703 were bought from American Type Culture Collection (ATCC). All the cell lines were cultured in the Roswell Park Memorial Institute (RPMI)‐1640 media containing 10% fetal bovine serum (FBS) (Gibco, NY), 100 U/mL penicillin, and 100 mg/mL streptomycin (Invitrogen, CA) in an incubator at 37°C with 5% CO_2_.

### RNA isolation and RT‐PCR analysis

Total RNA was extracted from frozen tissues using Trizol (Invitrogen) following the manufacturer's protocols. Both miR‐372‐3p and *FGF9* mRNA were reversely transcribed to cDNAs using the Reverse Transcription System Kit (Invitrogen). RT‐PCR was carried out following the instruction of SYBR^®^Premix EX Taq kit (TaKaRa, Japan). cDNAs were then amplified (primer sequences are listed in Table 1). *FGF9* mRNA expression was normalized to GAPDH expression and miR‐372‐3p to U6 snRNA, respectively. The relative expression of miR‐372‐3p and *FGF9* mRNA were calculated by 2^−∆∆C^_τ_ method.

**Table 1:** Sequence primers designed for qRT‐PCR

	Forward	Reverse
MiR‐372‐3p	5′‐TTTCACGACGCTGTAAACTCGCA‐3′	
FGF9	5′‐ GGGCCCCCCTGGTCCGTCCTA‐3′	5′ ‐ TTCTTTTATCTCTCTCTCTTT‐3′
U6	5′‐CTCGCTTCGGCAGCACA‐3′	5′‐AACGCTTCACGAATTTGCGT‐3′
GAPDH	5′‐ACAACTTTGGTATCGTGGAAGG‐3′	5′‐GCCATCACGCCACAGTTTC‐3′

### Cell transfection

The cells were seeded into 24‐well plates and transfected with miR‐372‐3p mimics, miR‐372‐3p inhibitors or miR‐negative controls (NCs) using Lipofectamine^™^ 2000 (Invitrogen) following the manufacturer's instructions. *FGF9* cDNAs were subcloned into the pLenti‐GIII‐UbC lentivirus vectors and transfected into cells. Empty lentiviral vectors were used as negative controls. Viral packages were harvested after 24 h's transfection. MiR‐372‐3p mimics, miR‐372‐3p inhibitors, miR‐NCs, and *FGF9* cDNAs were synthesized by GenePharma Co., LTD (Shanghai, China).

### Dual luciferase reporter gene assay

A wild‐type or mutated *FGF9* 3′‐UTR segment was inserted between the XhoI and PmeI restriction sites of the pMiRGLO vector (Sangon Co., LTD, ShangHai, China). The reconstructed vectors were then cotransfected with miR‐mimics or miR‐NCs into NCI‐H520 cells and incubated for 48 h. Ultimately, the activities of Firefly and Renilla luciferases were detected by Dual‐Luciferase Reporter Assay System (Promega, Madison, USA). The luciferase activities of firefly were normalized against Renilla to represent the relative luciferase activity of cells with different transfection.

### 3‐(4,5‐dimethyl‐2‐thiazolyl)‐2,5‐diphenyl‐2‐H‐tetrazolium bromide (MTT) assay

Incubated cells were harvested and cultured in 100 *μ*L fresh RPMI‐1640 medium at a density of 2000 cells/well. 20 *μ*L MTT solution (5 mg/mL) was added into each well once a day for days. After 4 h's incubation, the original culture solution was discarded and 200 *μ*L DMSO was added to dissolve the excessive crystals. Optical absorbance of cells was measured using a multifunctional microplate reader at 490 nm.

### Colony formation assay

Approximately 200 cells were seeded in a 24‐well plate and cultured in RPMI‐1640 supplemented with 10% FBS for 2 weeks. Then the media were removed and the colonies were washed twice with PBS. Ultimately, cells were fixed by 4% methanol, stained with Giemsa for 15 min. The number of naked‐eye‐can‐see colonies was counted under an optical microscope.

### Flow cytometry

After 48 h's transfection, cells were harvested and suspended. To detect cell cycle distribution, 5 *μ*L PI and 10 *μ*L RNase A were added to the cell suspension and then incubated for 30 min. FACSalibur flow cytometer (Becton‐Dickinson, CA) and Cell Quest software (BD Biosciences, CA) were used to analyze the cell cycle distribution.

For cell apoptosis analysis, 5 *μ*L PI and 5 *μ*L FITC‐Annexin V (Sigma‐Aldrich, St. Louis, MO) were added to cell suspension. FITC‐ Annexin V‐positive cells were considered apoptotic.

### Transwell assay

1×10^5^ cells were seeded into the Transwell inserts with 8 *μ*m‐pore membranes coated with Matrigel (Corning Incorporated, NY). Serum free media were added to the inserts, whereas 20% FBS media were added to the bottom chambers. Cells were then cultured for 24 h at 37°C. Cells on the upper surface of the membrane were removed and the rest of the cells were fixed with 4% methanol for 15 min, washed twice by phosphate‐buffered saline (PBS) and stained by crystal violet for 15 min. Invasive cells were observed under an optical microscope and the number of invaded cells in ten randomly selected 20× fields was counted.

### Western blot

Proteins were lysed using radioimmunoprecipitation assay (RIPA, Sigma‐Aldrich) lysate buffer and the protein concentrations were determined using the bicinchoninic acid assay (BCA) reagent kit (Beyotime, Jiangsu, China). Proteins in samples were separated using 10% sodium dodecyl sulfate‐polyacrylamide gel electrophoresis (SDS‐PAGE) (50 *μ*g/lane). The separated proteins were electro‐transferred onto polyvinylidene fluoride (PVDF) membranes, and subsequently incubated with nonfat milk for 2 h at the room temperature. The PVDF membranes were incubated with the primary antibodies (anti‐FGF9, 1:1,000, anti‐GAPDH, 1:1,000) at 4°C overnight. The intensity of protein bands was quantified using ImageJ software (NIH, USA).

### Statistical analysis

Statistical analyses were performed by SPSS19.0 (IBM, USA). The data were shown as mean ± standard deviation ($\overset{¯}{x}$±s). The Student's *t*‐test was used to analyze the significance of the difference between any two groups, whereas one‐way ANOVA was used among more than two groups. Kaplan–Meier analysis was performed to analyze the survival rates of patients with LSCC. Differences were regarded statistically significant if *P* values were less than 0.05.

## Results

### MiR‐372‐3p and *FGF9* expression in LSCC tissues and cell lines

**Figure 1:**
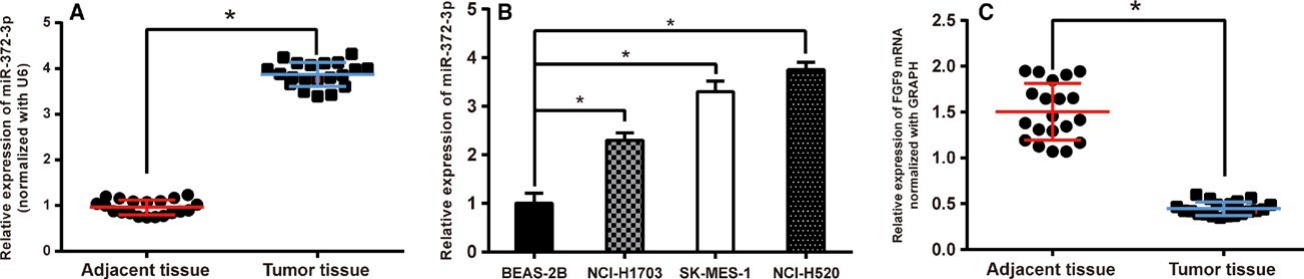
MiR‐372‐3p and *FGF9* levels in tissues or cell lines. (A) The relative expression level of miR‐372‐3p in LSCC tissues and adjacent tissues. (B) The relative expression level of miR‐372‐3p in different LSCC cell lines. (C) The relative expression level of *FGF9* mRNA in LSCC tissues and adjacent normal tissues. *P < 0.05.

We performed RT‐PCR to quantify the expression of miR‐372‐3p in LSCC tissues and adjacent tissues. As shown in Figure 1A–B, the results indicated that miR‐372‐3p was significantly upregulated in LSCC tissues and cell lines compared with adjacent tissues and normal cells, respectively (*P *< 0.05). We chose NCI‐H520 cell line for the subsequent experiments in vitro due to its highest miR‐372‐3p level.

On the other hand, *FGF9* mRNA expression was also determined by RT‐PCR. The relative expression level of *FGF9* mRNA was obviously lower in LSCC tissues compared with that in normal tissues (Fig. 1C; *P *< 0.05).

### *FGF9* was validated as a direct target of miR‐372‐3p

**Figure 2:**
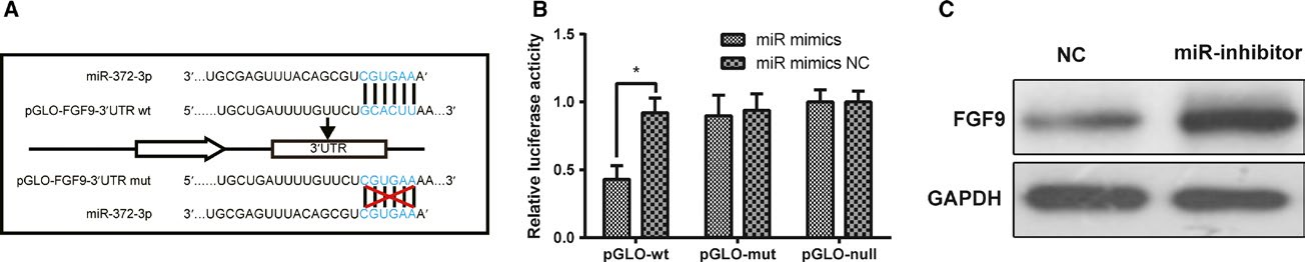
MiR‐372‐3p binding to *FGF9*. (A) The complimentary sequences on miR‐372‐3p, wild‐type *FGF9* 3′UTR, and mutated type *FGF9* 3′UTR. (B) Relative luciferase activity of NCI‐H520 cells. (C) Relative expression of FGF9 in LSCC cells transfected with miR‐372‐3p inhibitors or NCs. *P < 0.05.

Complimentary sequence of miR‐372‐3p on *FGF9* 3′UTR was obtained from a bioinformatics database, TargetScan (http://www.targetscan.org) and depicted in Figure 2A. To confirm that miR‐372‐3p could directly target *FGF9* 3′UTR, mutated *FGF9* 3′UTR (sequence showing in Fig. 2A) was also used in the following experiments. Dual luciferase reporter gene assay results validated the direct target relationship between miR‐372‐3p and *FGF9* (Fig. 2B). Briefly, the luciferase activity in wild‐type *FGF9* 3′UTR + miR‐372‐3p mimics subgroup was significantly lower than that in wild‐type *FGF9* 3′UTR + miR‐372‐3p NC subgroup. Whereas the luciferase activity in mutated *FGF9* 3′UTR + miR‐372‐3p mimics subgroup showed no difference from the that in mutated *FGF9* 3′UTR + miR‐372‐3p NC subgroup. Besides, we quantified the expression of *FGF9* protein in cells transected with miR‐372‐3p inhibitors by western blot. The results indicated that *FGF9* was obviously upregulated in cells that were transfected with miR‐372‐3p inhibitors (Fig. 2C, *P *< 0.05). Taken together, *FGF9* can be directly suppressed by miR‐372‐3p in LSCC cells.

### MiR‐372‐3p promoted LSCC cell viability and growth

**Figure 3:**
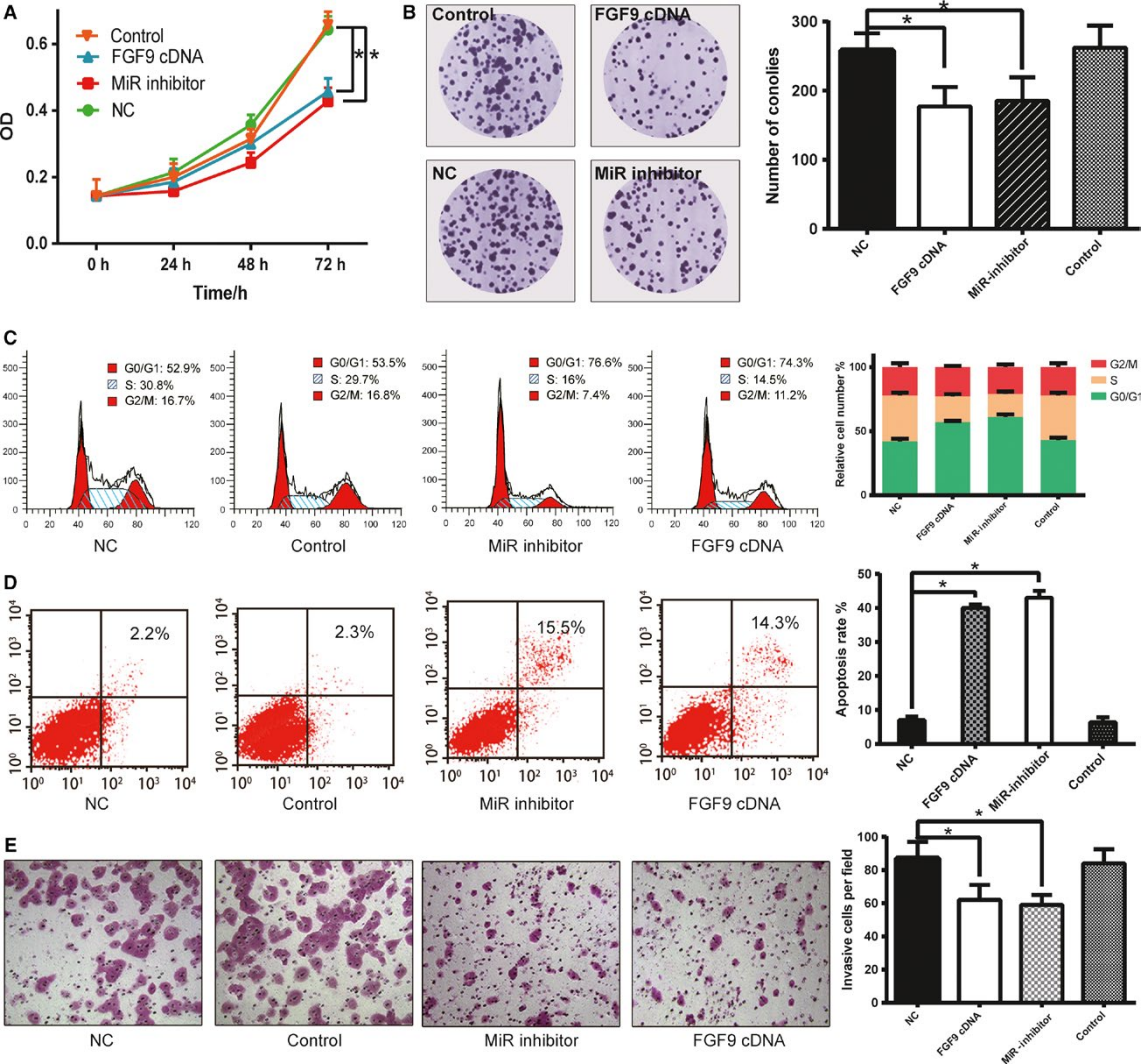
MiR‐372‐3p promoted LSCC cell growth and mobility. (A) The viability of NCI‐H520 in different groups. (B) The proliferation of NCI‐H520 cells in different groups. Flow cytometry results demonstrating cell cycle distribution (C) and apoptosis rates (D) of NCI‐H520 cells. (E) Transwell assay results showing LSCC the invasion of cells in different groups. All the experiments were individual and repeated for three times. *P < 0.05.

To explore the effects of miR‐372‐3p on LSCC cell growth, MTT and colony formation assays were performed on NCI‐H520 cells. The viability of cells transfected with miR‐372‐3p inhibitors were dramatically weaker than those transfected with NCs (Fig. 3A, *P < *0.0*5*). Meanwhile, the viability of those transfected with *FGF9* cDNAs was obviously suppressed compared with those transfected with NCs (*P < *0.0*5*).

Similarly, the colony formation results showed that the transfection of either *FGF9* cDNAs or miR‐372‐3p inhibitors led to the decreased cell proliferation (Fig. 3B).

### MiR‐372‐3p and *FGF9* ectopic expression inhibited LSCC cell mitosis and invasiveness

We transfected NCI‐H520 cells with miR‐372‐3p inhibitors or FGF9 cDNAs to see whether miR‐372‐3p and *FGF9* deregulation could affect NCI‐H520 cell mitosis. The cell cycle distribution and apoptosis rate of cells were analyzed. MiR‐372‐3p underexpression or *FGF9* overexpression induced cell cycle arrest in G0/G1 stage (Fig. 3C, *P *< 0.05). Likewise, the apoptosis rates of cells transfected with either miR‐372‐3p inhibitors or *FGF9* cDNAs were obviously higher than those transfected with NCs (Fig. 3D, *P *< 0.05).

To further detect the influence of miR‐372‐3p and FGF9 ectopic expression on LSCC cell invasiveness, we performed Transwell assay. As shown in Figure 3E, the repression of miR‐372‐3p led to the reduced LSCC cell invasion, suggesting that miR‐372‐3p could promote LSCC cell invasiveness (*P* < 0.05). Meanwhile, exogenous *FGF9* also substantially inhibited the LSCC cell mobility (*P* < 0.05).

## Discussion

Nowadays, with the deterioration of the environment, aggravation of the air pollution and prevalence of smoking, lung cancer gradually become a common cancer which severely threatens people's health and life. According to an investigation conducted by the GLOBOCAN project (http://globocan.iarc.fr/), the mortality and incidence rate of lung cancer were 16.7% and 23.2% respectively in 2012 and it has become the third most common cancer. Therefore, it is necessary and meaningful to explore the mechanism of tumorigenesis of the lung cancer.

MicroRNAs (miRs) have been widely accepted as a regulator of human cancers and it can be either cancer promoters or cancer suppressors. A plethora of studies have demonstrated that miR‐372 has a strong influence on some human cancers like gastric cancer, hepatocellular cancer, and colorectal cancer by regulating specific genes 29, 30, 31. MiR‐372‐3p, on the other hand, has not been reported to influence carcinogenesis too much, however, was discovered to suppress insulin‐like growth factor 2 mRNA‐binding protein 1 (IGF2BP1) therefore led to the decrease in proliferation and invasiveness in renal cell carcinoma 32. In this study, we explored the influence of miR‐372‐3p on lung squamous cell carcinoma (LSCC), and to explain the mechanism behind the effects, the targeting relationship between miR‐372‐3p and gene *FGF9* was also revealed.

Although miR‐372‐3p has been found to regulate cancer development by suppressing various genes in various tumors 32, 33, 34, it was worth mentioning that the *FGF9* was chosen as the candidate target gene using bioinformatics analysis. In this study, we found that miR‐372‐3p depressed the expression of *FGF9* directly. On the basis of the discovery that miR‐372‐3p was overexpressed and *FGF9* was underexpressed compared with adjacent tissues in three LSCC cell lines, we hypothesize that *FGF9* seemed to be the tumor suppressor and miR‐372‐3p might act as a tumor facilitator in LSCC.

*FGF9* belongs to the fibroblast growth factor (FGF) family that participates in a spectrum of biological processes including tumor growth and invasion. *FGF9* is specifically important in glial cell growth and development. It has been reported to suppress renal tumor metastases 35. However, it has also been discovered to facilitate gastric cancer invasion and growth 36. Besides, *FGF9* was believed to indicate a poor prognosis of non‐small‐cell lung cancer, colon cancer, and prostate cancer 15, 37, 38. However, we found that *FGF9* was underexpressed in LSCC tissues, which suggested its tumor suppressor role rather than a tumor facilitator role. The different roles of *FGF9* may due to the different context of different tumors, and further experiments are warranted to confirm its role in LSCC. *FGF9* was proved to be targeted by miRNAs such as miR‐155‐5p and miR‐140‐5p in lung dysplasia and hepatic cell carcinoma 39, 40. In our study, we also confirmed that *FGF9* could be directly suppressed by miR‐372‐3p, which contributed to the comprehensive understanding of LSCC carcinogenesis.

Taken together, exogenous miR‐372‐3p significantly suppressed *FGF9* expression, and simultaneously, LSCC cells *FGF9* can be directly repressed by miR‐372‐3p. This interaction between miR‐372‐3p and *FGF9* in LSCC led to the increase in tumor growth and invasiveness. We thus conclude that miR‐372‐3p could promote LSCC development by suppressing FGF9 expression.

Our research provided novel insight into the treatment of LSCC. Traditional treatments for LSCC are radiotherapy, chemotherapy, and lobe resection, which always do harm to patients and have a high risk of side effects and recurrence rates. Our study provides therapists a basis for further treatments of LSCC by targeting miR‐372‐3p or *FGF9*. However, some limitations remain in our study that *FGF9* is not the only gene that influencing the progression of LSCC, moreover, other miRNAs may regulate the expression of *FGF9* except for miR‐372‐3p in LSCC. Therefore, our future work will be dedicated to overcome the limitations, specifically, by investigating the relationship between other genes and the progression of LSCC.

In conclusion, the expression of miR‐372‐3p and *FGF9* in LSCC tissues and cells were determined and the effects of miR‐372‐3p and *FGF9* on LSCC cell activities have been studied. Besides, we confirmed the binding relationship between miR‐372‐3p and *FGF9*. So we come to that conclusion that miR‐372‐3p could probably promote LSCC growth and invasion by suppressing *FGF9* expression.

## Conflict of Interest

All authors declare no conflict of interest.
